# Artificial Agency as a New Force in Global Environmental Change

**DOI:** 10.1021/acs.est.6c09177

**Published:** 2026-08-06

**Authors:** Matthias C. Rillig

**Affiliations:** † 9166 Freie Universität Berlin, Institute of Biology, 14195 Berlin, Germany; ‡ Berlin-Brandenburg Institute of Advanced Biodiversity Research, 14195 Berlin, Germany

**Keywords:** global environmental change, climate change, chemical pollution, artificial intelligence, AI agents

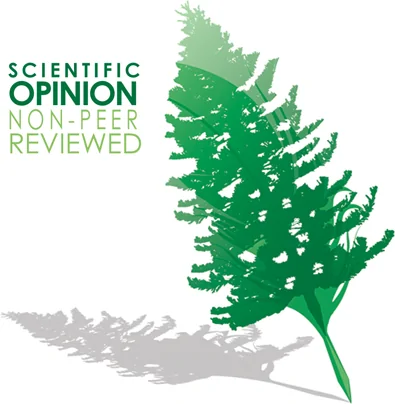

## Graphical abstract

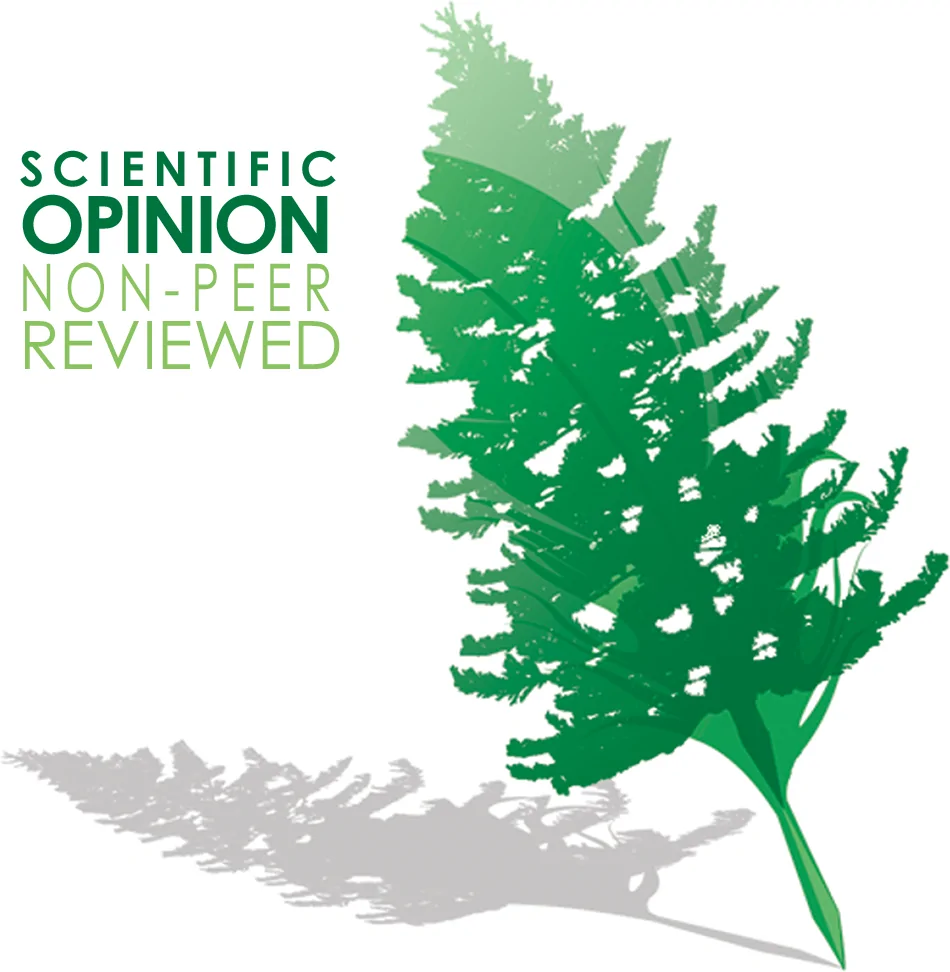

Global environmental change^1^ is typically defined as a human-caused phenomenon, encompassing factors that are global in scope and that affect living organisms.^2^ Human activity directly intervenes in ecosystems, as in land-use change (habitat conversion and overharvesting), or human actions lead to inadvertent consequences for ecological systems, such as invasive species, various forms of pollutants, and climate change (Figure 1).

**Figure 1:**
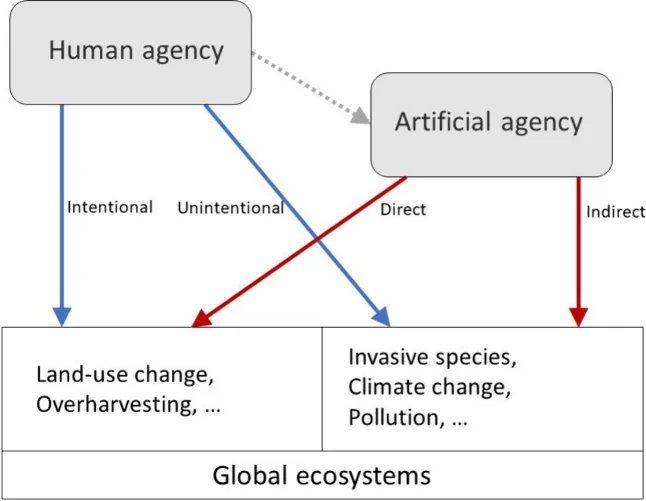
Human agency, as a result of intentional actions and unintentional consequences of activities, leads to various factors of global change. Artificial agents, acting autonomously, can have direct and indirect effects on ecosystems that are removed from human agency, constituting a new force to consider in global environmental change.

Even though global change is a highly complex, multidimensional issue,^3^ so far, a wide range of factors of global change all have their common origin in a range of human decisions and actions. For example, with human travel and transport of goods, biogeographical dispersal barriers for certain species are overcome, leading to the potential establishment of invasive species. As a consequence of activities, including cement production and fossil fuel burning, atmospheric concentrations of carbon dioxide have been increasing, or as a consequence of the widespread industrial production of certain chemicals, and their use in a variety of applications, certain persistent organic chemicals such as PFAS or particles of microplastic have accumulated in the environment.

However, the increasing development and implementation of artificial intelligence could mean that humans are no longer the sole root cause of factors of global change. A new kind of agency may become more apparent: artificial agency (Figure 1). An AI agent is an autonomous software system that can sense its environment, works through complex objectives, plans, learns, and adapts, and takes goal-directed actions (including spawning other AI agents) to achieve specific outcomes, all with minimal human intervention. The key distinction between AI agents and AI bots or assistants is their autonomous mode of action. While chatbots deliver output based on prompts, AI agents pursue multistep goals autonomously. We currently are witnessing only the beginning of the development and deployment of AI agents, and evolvable AI may arise as a future development,^4^ potentially representing even greater independence from human agency.

Environmental impacts of increasing AI use have already been well-documented^5^ (e.g., increased land, water, and electricity use by data centers), and this is certainly a cause for concern in a global change context. However, the point discussed here is much more fundamental. AI agents could be making decisions with minimal human oversight with potentially global environmental consequences. Among other consequences, discussed below, this non-human agency could also lead to increasing construction and use of AI data centers and thus greater environmental impacts from these.

## Direct and Indirect Effects of Artificial Agency

Artificial agents are in the end still designed and employed by humans. This means that effects can be traced back to humans, and thus, the classical definition of global change as a human-caused phenomenon still holds. However, compared to all other root causes of global change factors, human decisions and actions themselves are at least several steps removed from potential effects, replacing them with machine-made decisions and actions. It is this observation that warrants an examination of AI agency as a new (future) force in global change.

Artificial agents could affect ecosystems either directly or indirectly (Figure 1). When AI agents are put in charge of managing key aspects of certain ecosystems in the future, they could directly influence dynamics within the target system by affecting management interventions on the ground. This transition to full machine control is unlikely to happen for all ecosystems, but some ecosystems that are already under tight management, such as agricultural ecosystems, certain constructed urban ecosystems, or plantations, are candidates for higher degrees of control by artificial agents compared to more natural systems. For instance, in future agroecosystems, AI agents could control robotic weed removal, watering regimes, precision additions of fertilizer, and pesticide use, all based on extensive data streams from sensors and autonomously pursuing the singular high-level goal of maximizing profit, while placing lower weights on soil health and pollinator biodiversity. In urban systems, one could envision AI agents autonomously making decisions on control of invasive species, prioritizing removal of plant species whose pollen cause allergic reactions in humans. The transition of control will likely happen very gradually, starting from expert systems providing advice to human land managers that are still in control, and potentially resulting in autonomous control by AI agents, progressively reducing human intervention.

Indirect effects are very likely much more pervasive, potentially affecting all ecosystems, and diffuse and are very difficult to predict at this time. Indirect effect here means that AI agents make decisions, and these decisions have unforeseen, far-reaching effects on ecosystems. A potential positive example could be AI agents controlling automobile traffic, leading to reduced production of greenhouse gases and potentially reduced emission of tire-wear particles. A negative example could be AI agents pushing the use of certain chemicals or groups of chemicals because of their favorable properties for consumers, leading to an accumulation of such chemicals in the environment with problematic long-term consequences. In general, such indirect effects stem from AI agents pursuing certain high-level goals (such as maximizing profit and increasing the flow of traffic) without hard-wired incentives or training for avoiding negative effects, for example, on biodiversity or ecosystem health.

Artificial agency most likely does not give rise to new factors of global change; at least we do not know at this time. What will likely change with more prominent artificial agency are the trajectories, the intensity, and the combinations of factors. Artificial agents, by acting in real time, and by accessing unprecedented amounts of data, and by basing decisions on certain goals to be optimized, would arrive at decisions different from those of humans. The result could be a new spatial and temporal mosaic of environmental change across Earth.

## Risks, Opportunities, and the Need to Act

There are unique risks of AI that may lead to changes in how global change unfolds,^5,6^ and these also apply to agentic AI systems; these risks include cybersecurity issues, proprietary codes, goal conflicts, and a widening digital divide across different parts of the world. On the other hand, environmental stewardship could improve with AI agents trained on large amounts of data,^7^ making them more effective than current efforts,^8^ thus potentially mediating the effects of drivers of global change. Coupled with excellent monitoring and proper model training, a system based on AI agents could respond faster, based on more evidence, and thus better than currently feasible. These opportunities to change dynamics for the good exist and should be decisively supported; however, if profit and power continue to be major factors, there is perhaps less reason for optimism.

We are now only at the beginning stages of such non-human agency directly or indirectly influencing ecosystems. However, artificial agents are becoming pervasive in their integration into many areas of human affairs.^9^ This very nature of pervasive integration poses formidable and unprecedented challenges in terms of governance, jurisdiction, training, accountability, and policy.

Now is the time to address fundamental questions. The first is how much direct control over ecosystems we should yield to AI agents in terms of direct management decisions. It may be necessary to conclude that there will always have to be some degree of human oversight when ecosystems stand to be directly impacted. The second important topic pertains to the more diffuse, indirect influence that agentic AI will have on who is held accountable for agentic AI-related changes in nature that arise as a consequence of AI agent decisions, and what ethical, legal, and technical safeguards should be implemented to avoid adverse effects. A prime target for such interventions concerns system prompting, model coding, training, and verification. AI agents active in diverse fields like healthcare, traffic, manufacturing, and other realms will need to be properly trained on data and instructed; no-go areas need to be specified, and agents need to be tested for compliance in predeployment simulations. In addition, there ideally needs to be run-time gatekeeping while the agent is active, and AI agent decisions will require post hoc audits after the fact. It is very difficult to predict the consequences of many AI agents interacting; this will also need to be included in risk assessments. The key issue is in governance: who will enforce this set of safety checks for a diverse range of AI agents designed by different corporations in different nations in a race to produce the most desirable AI agent for the market. IPBES^10^ and other intergovernmental bodies concerned with the environment and national and local organizations will need to put this new force on their agendas. It will be critical to stay a step ahead of the upcoming rapid developments and to put pressure on governments to act, exploring levers such as market access, financial incentives (e.g., insurance), and others, thus helping to unlock the benefits and to avert negative consequences of greater AI agential influence on the environment at a global scale.
